# An interaction of a NR3C1 polymorphism and antenatal solar activity impacts both hippocampus volume and neuroticism in adulthood

**DOI:** 10.3389/fnhum.2013.00243

**Published:** 2013-06-05

**Authors:** Christian Montag, Markus Eichner, Sebastian Markett, Carlos M. Quesada, Jan-Christoph Schoene-Bake, Martin Melchers, Thomas Plieger, Bernd Weber, Martin Reuter

**Affiliations:** ^1^Department of Psychology, University of BonnBonn, Germany; ^2^Center for Economics and Neuroscience, University of BonnBonn, Germany; ^3^Department of Psychology, University of GiessenGiessen, Germany; ^4^Department of Epileptology, University of BonnBonn, Germany; ^5^Department of NeuroCognition/Imaging, Life and Brain CenterBonn, Germany

**Keywords:** NR3C1, solar activity, personality, structural MRI, pregnancy

## Abstract

The investigation of the interaction of genes and environment in the context of mental health and personality yields important new insights for a better understanding of human nature. Both antenatal and postnatal environmental factors have been considered as potential modulators of genetic activity. Antenatally, especially smoking or alcohol drinking habits of the mother dramatically influence the health of the child during pregnancy and even later on in life. In the present study we would like to introduce a more “distant” factor that is not under the control of the becoming mother but that nevertheless plays a potential role for the health of the unborn child later on in adulthood. Here, we retrospectively investigate the influence of solar activity (while the child is still in the uterus of the becoming mother) on brain structure (with a focus on hippocampus and amygdala volume) and personality in adulthood. We observe an interaction of a genetic variant (rs41423247) of the glucocorticoid receptor gene (NR3C1) and solar activity in the first trimester after conception on both hippocampal volume and the personality trait neuroticism in adulthood in *N* = 254 participants. The NR3C1 gene is the focus of interest, because of its influence on the hypothalamic-pituitary-adrenal (HPA) axis and negative emotionality. Carriers of the CC variant of rs41423247 grown in the womb under the influence of high sun radiation (high solar activity) show both the highest hippocampal volume in the left hemisphere and lowest neuroticism scores. The present findings should encourage researchers in psychology and psychiatry to include also environmental influences such as solar activity besides genetics to better understand the etiogenesis of psychiatric disorders.

## Introduction

Psychiatric conditions are both the cause for great individual suffering and for high costs to society. In order to better understand how psychopathological disorders form, both genetic and environmental factors have been considered to be of importance (see a recent review by Cohen-Woods et al., 2012). In the last years an important research focus has been to disentangle how environmental influences shape genetic activity. This has been done by the investigation of gene by environment effects on psychiatric conditions such as depression (Caspi et al., 2003) or more directly by the investigation of environmental factors shaping the methylation pattern of genes i.e. epigenetics (see review by Caldji et al., 2011).

In the context of environmental vulnerability factors for psychopathological conditions a large number of postnatal factors have been considered ranging from dietary issues (Haleem, 2012), smoking (Edwards and Kendler, 2012) and alcohol consumption (Flensborg-Madsen, 2011) to other forms of addictions including non-substance dependent addictions (e.g., Gupta et al., in press). In the last years a growing interest has led to a shift, whereas not only postnatal but in particular also antenatal (and perinatal) factors have become a focus of research in order to understand how environmental factors during fetal gestation influence the unborn child and shape its later life ranging from birth to adolescence/adulthood. Besides obvious negative factors such as alcoholism (Paintner et al., 2012a,b; Popova et al., 2012) or smoking habits (Liu et al., 2011; McDonnell-Naughton et al., 2012) of the expectant mother also states of anxiety, depression and stress during pregnancy have been shown to influence variables such as preterm birth or low birth weight and also a vast range of cognitive functions later on in the life of the still unborn child (see review by Dunkel Schetter and Tanner, 2012). Parts of the investigated antenatal environmental factors causing psychopathological conditions after birth are more or less under the control of the mother (such as smoking or alcohol consumption) and postnatally especially in adolescence and adulthood under the control of the person afflicted with a psychiatric condition. As a consequence, these factors have been abundantly investigated as they are at first sight of highest relevance, because behavioral modification strategies can help to prevent or overcome psychiatric conditions.

The present study follows a new path by investigating a factor not under control of the becoming mother, which nevertheless might shed new light on the development of depression and anxiety disorders. Here, besides a genetic factor being involved in the etiogenesis of negative emotionality (to be introduced later on) the intensity of cosmic rays in form of solar activity during pregnancy will be considered as a physical factor shaping the health of the yet unborn child later on in adolescence. How can this be of importance? The sun is essential for life on earth. It provides the necessary energy for various chemical processes including photosynthesis and, in combination with the atmosphere, the temperatures to establish and maintain higher life forms. A wide-range spectrum of high-energy particles, caused by coronal mass ejections, sunspots, solar flares and the constant solar wind, is emitted towards the earth. A broader term for this is solar activity. The solar activity is highly correlated to the number of sunspots visible on the solar disk. They follow a cyclic pattern of about eleven years from one minimum of solar activity to the next. The relative sunspot number R^1^ can exceed 250 in highly active periods (maxima) of the sun (Moldwin, 2008). Sunspot cycles vary in length and intensity. During a cycle the relative sunspot number R can range from 0 at a minimum to over 250 (Harvey and Wilson, 2000). The energetic particles from the sun interact in various ways with the ionosphere, e.g., the aurora borealis, the geomagnetic field of the earth and electronic equipment. Solar activity has been linked to earth climate (Rind, 2002), to human behavior and health risks (for a review see Palmer et al., 2006) and even economic decision-making (Kliger et al., 2012). In a similar context, it has been hypothesized by Davis and Lowell (2004) that the occurrence of mental disorders might be influenced by changes in the ultraviolet radiation (UVR) during solar cycles. In addition, several authors report a negative impact on the human cardiovascular system including higher mortality rates in periods of higher solar activity (Malin and Srivastava, 1979; Cornélissen et al., 2002; Stoupel et al., 2011). Halberg et al. (2000) report influences on anthropometric morphological variables such as neonatal body heights, body weight and also head circumference. A study by Gortmaker et al. (1997) provided evidence for a link between short daylength in the middle of gestation and shyness in childhood. These findings imply possible antenatal influences of solar activity on a cellular level. Furthermore, Zaporozhan and Ponomarenko (2010) showed altering influences of weak magnetic fields and their fluctuations, caused by solar activity, on expression of genes related to the function of NF-κB and other biological regulators, e.g., hormones. NF-κB represents a transcription factor, which is common in most of the cells. Of particular interest for the present study, Zaporozhan and Ponomarenko (2010) report that magnetic fields are able to modulate glucocorticoid pathways via a complex protein cascade including proteins from the CRY family (Cryptochrome). An influence of magnetic storms elicited by the sun on the circadian (24 h) corticosterone rhythm has been shown in rats by Jozsa et al. (2005).

Given these first studies on solar activity and human conditions, we investigated if a well-known genetic vulnerability factor for negative emotionality combined with high or low solar activity in pregnancy influences the volume of the brain structures hippocampus and amygdala in adolescence. Mounting evidence suggests that these brain structures are associated with lower gray matter volume in depressed patients compared to control groups (Sheline, 2011; Bora et al., 2012). Knoops et al. (2010) linked lower hippocampus volumes to higher cortisol levels in the evening and to a lower suppression of cortisol after a dexamethasone-test, which might outline one of the hormonal pathways involved in depressive behavior. Lower hippocampus volumes have been shown for anxious healthy participants, too (Yamasue et al., 2008). In particular, one of the most prominent theories on the neuropsychology of anxiety (Gray and McNaughton, 2000) explicitly stresses the central importance of hippocampus and amygdala in a large anxiety network. Adding to the investigation of human brain structure, we searched for an influence of a genetic variant related to stress responsivity (the BCL1 polymorphism on the NR3C1 gene, see below) and solar activity on the personality dimension neuroticism. Neurotic persons tend to be moody, have higher feelings of guilt and are emotionally instable. It has been demonstrated that neuroticism represents an important vulnerability factor for the development of depression and anxiety disorders (Kendler and Myers, 2010).

The present study not only tries to establish the link between genes, solar activity, brain structure and personality, but also tries to relate the personality dimension neuroticism directly to the mentioned brain structures located in the temporal lobes of the human brain. The effects of genetics and environment via brain structure on personality are investigated. On the genetic side we target a well-known genetic candidate in the context of depression research related to the hypothalamic-pituitary-adrenal (HPA) axis: One of the most prominent biological theories of depression hypothesizes that a dysregulation of the HPA axis is one of the causes for affective disorders (Holsboer and Ising, 2008). Here, the major hormone of interest is cortisol. In the regulation of the HPA-axis cortisol uses negative feedback loops to downregulate the cortisol production in the adrenal glands (if the endogenously produced cortisol levels are too high). One important element in this negative feedback loop represents the glucocorticoid receptor, as it represents a prominent binding site for cortisol on neurons. The glucocorticoid receptor is encoded by the NR3C1 gene, which is located on chromosome 5q31–5q32. An interesting candidate for the present study represents the single nucleotide polymorphism (SNP) rs41423247 (also known as BCL1 polymorphism), which can be found on intron B (647 kb downstream of exon 2; Derijk, 2009). It is of interest, because it has been shown to be associated with morning cortisol suppression in the dexamethason test (Wüst et al., 2004) and to be a modulator of glucocorticoid receptor sensitivity (Huizenga et al., 1998; Stevens et al., 2004). Van Rossum et al. (2006) reported that the GG genotype occurred significantly more often in patients with major depression in contrast to controls. Recently, Reuter et al. (2012) reported an interaction effect with a combination of the CC variant (or, alternatively, labeled the G− variant) together with the TT variant of a prominent mutation on the cholinergic CHRNA4 gene (rs1044396) as a risk factor for depression. This study shows that effects are more complex when interactions between gene loci are investigated. Nevertheless, the findings by Van Rossum et al. (2006) and Reuter et al. (2012) seem at first glance contradictory. To add to the confusion, Van Rossum et al. (2003) found an impaired negative feedback loop of the HPA-axis in CC carriers as indicated by increased cortisol levels after a dexamethasone-test. Normally increased cortisol levels after the dexamethasone-test—as observed in CC carriers—are an indicator of depression.

In sum, this study investigates the interaction of the NR3C1 gene and solar activity on both brain structure and personality. Based on the above mentioned findings, namely an association between the GG genotype and depression (Van Rossum et al., 2006), neuroticism being a risk factor for depression (Kendler and Myers, 2010), and reduced hippocampus volume in depressive patients (Sheline, 2011), we hypothesized that carriers of the CC (i.e., G−) variant of rs41423247 together with high or low solar activity^2^ during fetal gestation are related to lower neuroticism scores and higher volume of hippocampus/amygdala. We expected the effects to be highest in the first trimester after conception, because a recent review by Dunkel Schetter and Glynn (2011) provided evidence that stressors such as major life events impact preterm birth strongest in the early phase of pregnancy.

## Methods

### Participants

The sample consisted of 71 males and 183 females (*N* = 254 Caucasian participants). More females than males participated, because most participants were recruited from psychology classes (with higher females studying psychology). Mean age was 25.57 (*SD* = 8.30). All participants were healthy and free of any neurological/psychopathological disorder. All participants underwent structural imaging at 1.5T (*n* = 131) or 3T (*n* = 123), were asked for their birthdate and provided buccal cells for the genetic analysis. Moreover the participants filled in the revised Eysenck's Personality Questionnaire (EPQ-R) to measure neuroticism (Eysenck and Eysenck, 1991). The study was approved by the medical ethics committee of the University of Bonn, Germany. This sample has recently been analyzed in parts in the context of volumetric hemispheric ratio and personality by our own group in another study (Montag et al., 2013a).

### Measurement of solar activity

Solar activity was inferred from data of the international sunspot numbers database^3^, containing relative sunspot numbers since 1818. Gestation is commonly divided in trimesters, which we considered to last 89 days each. Therefore, the hypothetical day of conception was 267 days before birth, exceeding the reported period in the literature by one day, for trimester convenience (see e.g., Saladin, 2007). Average sunspot numbers were calculated for each subject's trimesters during gestation ranging from 1.20 to 236.91 in the sample (M_M1−3_ = 77.72, SD_M1−3_ = 56.36). Average sunspot numbers were calculated for each subject's trimesters during gestation. No mean differences in sunspot numbers between men and women were observed in the three trimesters (*F*-values <1, n. s.; here three ANOVAs have been calculated with average sunspot numbers of the respective trimester as dependent variable and sex as the independent variable). A median split for each trimester was performed to assign subjects into low or high solar activity groups. A limitation of this approach to be mentioned represents among others the loss of information after dichotomization (MacCallum et al., 2002). Despite this, in our study the median split could be justified by the idea that solar activity itself might influence epigenetics in form of high or low activity (e. g., it is imaginable that a certain threshold needs to be reached in order to see an impact on a molecular level).

### Measurement of personality

The personality dimension neuroticism was measured by means of EPQ-R (Eysenck and Eysenck, 1991) a self-report measure to assess personality on the three dimensions psychoticism, extraversion and neuroticism. The neuroticism scale which is of relevance for the present study consists of 25 dichotomous items to be answered with “yes” or “no.”

### Genetic analysis

DNA was extracted from buccal cells. Automated purification of genomic DNA was carried out with the MagNAPure LC system using a commercial extraction kit (MagNA Pure LC DNA isolation kit; Roche Diagnostics, Mannheim, Germany). Genotyping of the rs41423247 (BCL1) polymorphism was performed by real-time PCR using fluorescence melting curve detection analysis by means of the Light Cycler System (Roche Diagnostics). By means of the melting curve analyses, SNPs can be detected without conducting gel electrophoresis or sequencing after amplification. The primers and hybridization probes (TIB Molbiol, Berlin, Germany) and the PCR protocol can be found in Reuter et al. (2012).

### Magnetic resonance imaging

The scanning protocol used for the morphological analysis included a T1-weighted 3D sequence, acquired either on an Avanto 1.5 Tesla or Trio 3 Tesla MRI scanner (both manufactured by Siemens, Erlangen, Germany). We used an MP-RAGE sequence with 160 slices (*TR* = 1300 ms, *TI* = 650 ms, *TE* = 3.97 ms, resolution 1.0 × 1.0 × 1.0 mm, flip angle 10°). Sequence parameters, size of the matrix and acquisition time were identical for the two scanners. Sex distribution differed between participants scanned at 1.5T or 3T (χ^2^ = 16.73, *df* = 1, *p* < 0.001). Therefore, we also tested for sex and scanner effects on the recorded brain volumes.

T1-weighted raw images were converted into NIfTI-1 format and processed with FSL (Version 4.1, FMRIB Analysis Group, University of Oxford, England). Subcortical structures, including amygdala and hippocampus, were segmented using a model-based registration and segmentation tool (FIRST) as described by Patenaude et al. (2011). This method uses models from different subcortical structures constructed from manual segmentations of 336 subjects and fits them to the brain of the subject being analysed by using a bayesian approach for shape and intensity. This process differs from the usual intensity-dependant tissue classification methods like traditional voxel-based morphometry, which may deliver worse results in subcortical areas being more prone to imaging artifacts. Volume from the extracted hippocampi and amygdalae of each subject was then used for the statistical correlations with the genetic and personality variables. For statistical analyses, whole brain volume was used as a covariate.

### Conditional effects model

Indirect effects can be observed in mediation models. Here, the impact of a predictor is partially or totally mediated on the criterion by a third variable (the mediator). The findings in the result section encouraged us to test a moderated mediation model. The classic perspective on mediation models (Baron and Kenny, 1986) requires a correlation among all three mentioned variables (predictor, mediator, criterion). Adding to this, Hayes (2009) demonstrated that indirect effects are also possible to appear in the absence of a correlation between predictor and criterion.

To assess indirect effects between our variables (effects from genes via brain structure on personality) we used PROCESS for SPSS (Hayes, 2012, version B130612). The moderated mediation model we used for our findings is specified in PROCESS as model 21. For this analysis it is not possible to estimate an effect size of the conditional indirect effect. Our model assumes moderators on path *a* (the relation between gene and hippocampus volume is moderated by solar activity) and *b* (the relation between hippocampus volume and neuroticism is moderated by sex). Please see Figure 5. The conditional indirect effect of *X* (genetic variation on the gene NR3C1) on *Y* (the personality dimension neuroticism) through the mediator (brain structure) is defined as the product of the conditional effects of the moderations^4^. Hippocampus volume was regressed on the whole brain volume and neuroticism on age, using the resulting standardized residuals in the model instead of entering both as covariates. PROCESS provides a bootstrap option as a resampling method to statistically back up indirect effects. This method is more robust than others (see e.g., MacKinnon et al., 2004). Bootstrap samples were set to 1000 and confidence intervals were bias corrected. Both moderators were dichotomous. Therefore, indirect effects refer to groups of participants, either male or female with high or low solar activity. The predictor of the model, gene variant, was dichotomous (G±), too. In this case *B* indicates for which gene variant the hippocampus mediated the effect between gene and neuroticism.

## Results

### Age, sex, and neuroticism

Women showed significantly higher neuroticism scores compared to men [*t*_(252)_ = −2.43, *p* = 0.02]. Age correlated significantly with neuroticism (*r* = −0.24, *p* = 0.0001). Both sex and age were controlled for in the following statistical analyses of neuroticism.

### Age, sex, MRI scanner type and volume of hippocampus and amygdala

#### Age and volume of hippocampus and amygdala

Age correlated significantly with both the right hippocampus (*r* = 0.17, *p* = 0.008) and the right amygdala volume (*r* = 0.18, *p* = 0.004) while controlling for whole brain volume. Therefore, we considered age as a covariate in the following analyses when dealing with the right hemispheric structures. No correlations could be observed for the analyses of the left hippocampus/amygdala and age. Therefore, there was no requirement to control for age in the analyses of both left hippocampus and amygdala.

#### Sex, MRI scanner type and volume of the hippocampus and the amygdala

Multivariate analysis of covariance (MANCOVA) for the left hemisphere (including left hippocampus and left amygdala) with whole brain volume as a covariate and both sex and MRI scanner type as independent variables revealed a larger left amygdala volume in males compared to females [*F*_(1, 249)_ = 4.89, *p* = 0.03]. No significant differences could be observed for the left hippocampus volume. Moreover, no significant effect of MRI scanner type or MRI scanner type by sex could be observed on both the left hippocampus and amygdala volume.

A MANCOVA for the right hemisphere (including right hippocampus and right amygdala) with whole brain volume and age as covariates revealed a larger right amygdala volume in males compared to females [amygdala: *F*_(1, 248)_ = 11.73, *p* = 0.001]. No significant effect for the right hippocampus volume could be observed. Moreover, no significant effect of MRI scanner type or MRI scanner type by sex could be observed on both the right hippocampus and amygdala volume.

### Genotype distribution

The genotype distribution was as follows: *GG* = 106, *GC* = 107, *CC* = 41. This distribution is in Hardy Weinberg Equilibrium (HWE; χ^2^ = 2.46, *df* = 1, n. s.). The male subsample is in the HWE, too (*GG* = 28, *GC* = 36, *CC* = 7; χ^2^ = 0.88, *df* = 1, n. s.). In contrast the female subsample was not in HWE (*GG* = 78, *GC* = 71, *CC* = 34; χ^2^ = 5.70, *df* = 1, *p* < 0.05). In this context we would like to mention two things. First, in our opinion the HWE deviation in the female subsample is not of importance for our main results, because the total sample (where the following effects are reported for) is in HWE. Moreover, the large gene data bank (including *N* = 1068 where the participants of this study were drawn from) do not deviate from HWE in either the total or male/female subsamples. We conclude that the deviation from HWE in our female subsample happened by chance. As the independent variables in the present study represented the allelic variants G± and solar activity (high/low), we report the subsample size for each cell in Table 1.

**Table 1:** **Interaction effect of rs41423247 (G±) by solar activity in the first trimester (high/low) on the left hippocampus volume (means and SEM are presented)**.

	**High solar activity**	**Low solar activity**
rs41423247 G+	3937.66 (34.27)/*n* = 108	3994.02 (34.75)/*n* = 105
rs41423247 G−	4126.40 (79.63)/*n* = 20	3844.75 (77.72)/*n* = 21

All presented values are controlled for whole brain volume.

### Main effects of rs41423247 (G±) on the brain volumes of hippocampus and amygdala

No significant effects of rs41423247 (G±) could be observed on the hippocampus and amygdala volumes. No gene by sex effects could be observed on both brain structures.

### Solar activity (high or low) in all trimesters before birth and brain volumes of the hippocampus and amygdala

MANCOVAs with hippocampus and amygdala volumes as dependent variables and solar activity (high/low) as independent variable (calculated separately for each trimester) and whole brain volume and age as covariates were computed. A significant statistical result appeared for the analysis of the right amygdala volume with the independent variable solar activity (high/low) in the second [*F*_(1, 254)_ = 3.94, *p* = 0. 048] and third trimester [*F*_(1, 254)_ = 4.04, *p* = 0.046]. Higher solar activity was associated with higher amygdala volumes. A further MANCOVA considering sex as a further independent variable revealed no interaction effects with solar activity on amygdala volumes. This supplementary analysis was conducted only with amygdala volumes as dependent variable, because the hippocampus volumes were not influenced by sex (see above).

### rs41423247 (G±) by solar activity (high/low) interaction on the brain volumes of hippocampus and amygdala dependent on the respective trimester

**Figure 1:**
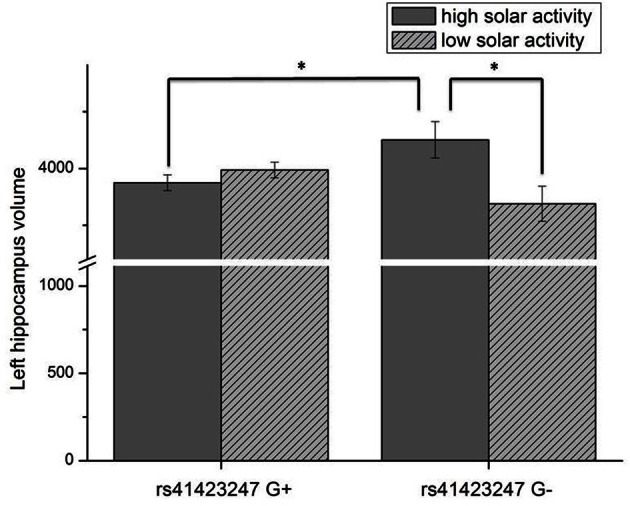
Interaction effect of rs41423247 (G±) by solar activity in the first trimester (high/low) on the left hippocampus volume (means and SEM are presented; ^*^p < 0.05).

**Figure 2:**
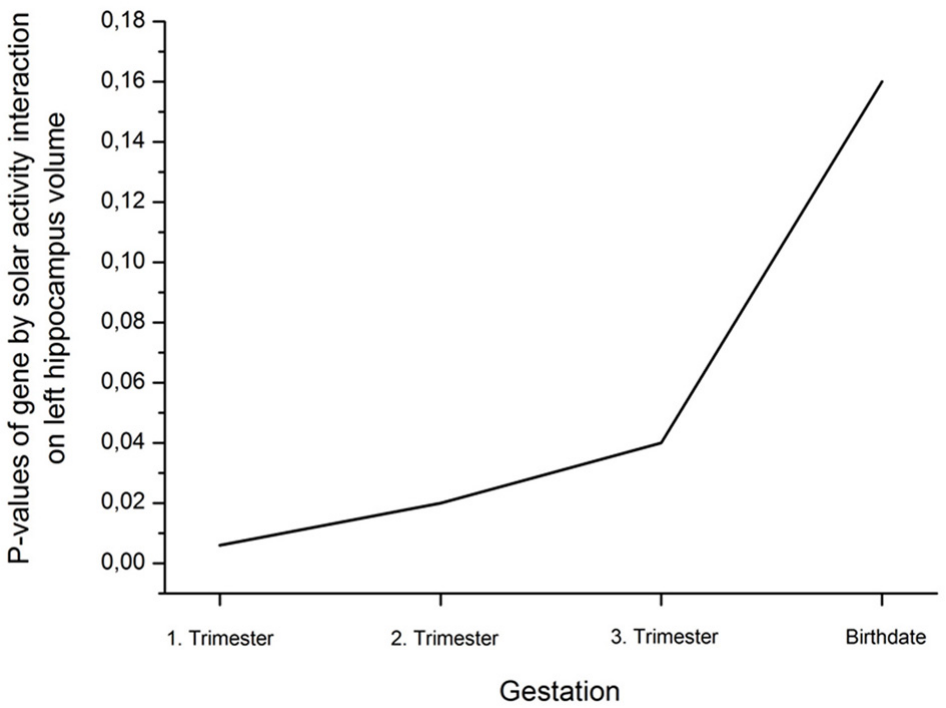
**Decline (rise of *p*-values; η^2^ are presented in Table 2) of the interaction effect gene by solar activity from the first trimester to birth on left hippocampus volume**.

Analysis of covariance (ANCOVA) with whole brain volume as covariate revealed a significant rs41423247 (G±) by solar activity (high/low) interaction effect for the left hippocampus in the first trimester [*F*_(1, 249)_ = 7.74, *p* = 0.006]. In detail, post hoc tests revealed that carriers of the G− variant of rs41423247 exposed to the highest solar activity during the first trimester in the womb of the mother showed significantly the highest volume of the left hippocampus compared to two of the remaining three groups (*p* < 0.05). All results are presented in detail in Table 1 and Figure 1. See Table 2 for the statistical results of all trimesters with respect to the left hippocampus. A decline of the effects towards birth can be observed. This is depicted in Figure 2. Besides these mentioned effects on the left hippocampus no other significant results can be reported.

**Table 2:** **Decline of the interaction effect gene by solar activity from the first trimester to birth on left hippocampus volume (η^2^ are partial η^2^)**.

1. Trimester of gestation	*F*_(1, 249)_ = 7.74, *p* = 0.006; η^2^ = 0.030
2. Trimester of gestation	*F*_(1, 249)_ = 5.32, *p* = 0.02; η^2^ = 0.021
3. Trimester of gestation	*F*_(1, 249)_ = 4.30, *p* = 0.04; η^2^ = 0.017
Birth date	*F*_(1, 249)_ = 2.03, *p* = 0.16; η^2^ = 0.008

### rs41423247 (G±) by solar activity (high/low) interaction in all trimesters on neuroticism

**Figure 3:**
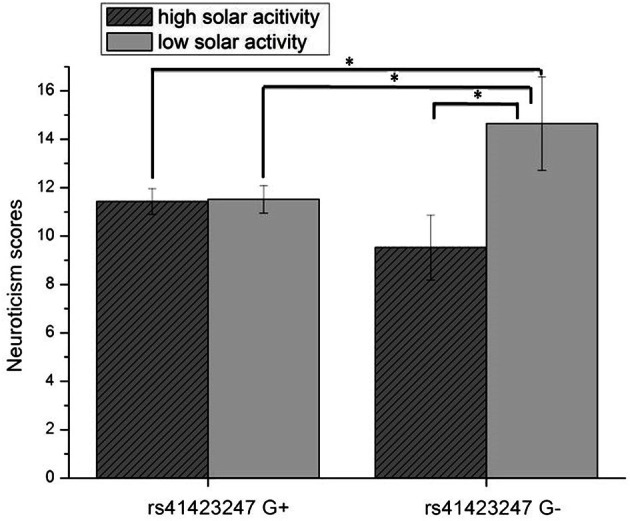
Interaction effect of rs41423247 (G±) by solar activity in the first trimester (high/low) on neuroticism (means and SEM are presented; ^*^p < 0.05).

**Figure 4:**
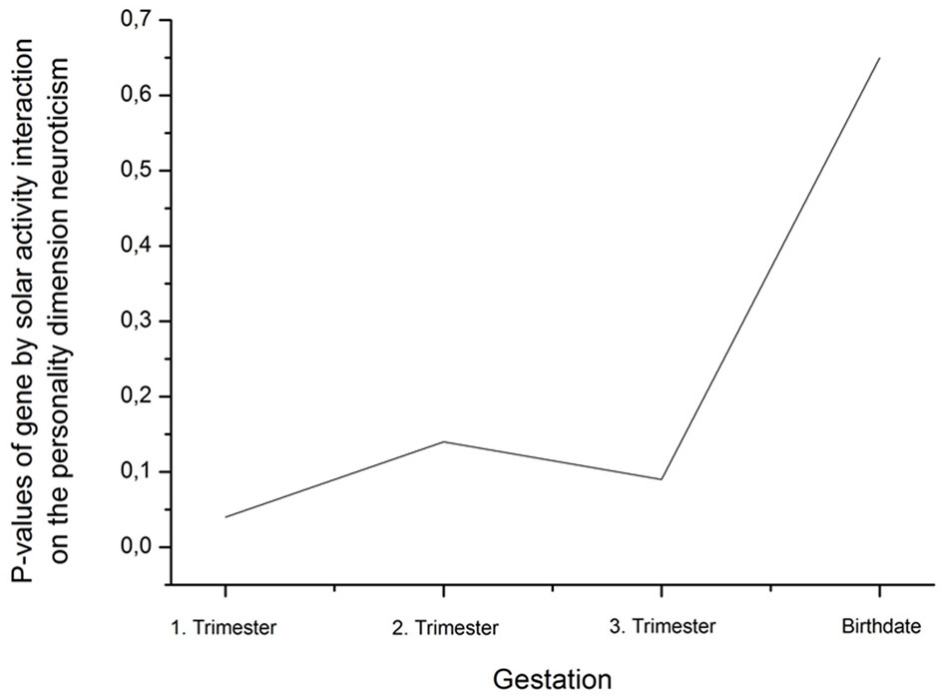
**Decline (rise of *p*-values) of the interaction effect gene by solar activity from the first trimester to birth on the personality dimension neuroticism (η^2^ are presented in Table 4)**.

An ANCOVA with age as covariate revealed a significant rs41423247 (G±) by solar activity in the first trimester (high/low) interaction effect on neuroticism [*F*_(1, 245)_ = 4.14, *p* = 0.04]. In this statistical analysis sex (male/female) was included as a further independent variable given its reported influence on neuroticism. No interaction effects between sex and rs41423247 (G±) or between sex (male/female) and solar activity (high/low) on neuroticism was observed. Moreover, no three way interaction between rs41423247 (G±), solar activity (high/low) and sex (male/female) on neuroticism could be detected. This analysis is not very meaningful, because the cell sizes are very small. In detail, carriers of the G− variant of rs41423247 exposed to the highest solar activity in the womb of the mother had the lowest neuroticism scores. A *post hoc* test revealed that the neuroticism scores of this group were significantly smaller than of those with the configuration G–/low solar activity (*p* < 0.05). No significant differences could be observed between G–/high solar activity group and the other two remaining configurations. The group G–/low solar activity exhibiting the highest neuroticism scores differed significantly from all three other groups (*p* < 0.05). All results (including the analysis for the other trimesters) are presented in detail in Tables 3, 4 as well as in Figures 3, 4. No other effects could be observed.

**Table 3:** **Interaction effect of rs41423247 (G±) by solar activity in the first trimester (high/low) on neuroticism (means and SEM are presented)**.

	**High solar activity**	**Low solar activity**
rs41423247 G+	11.43 (0.53)/*n* = 108	11.52 (0.57)/*n* = 105
rs41423247 G−	9.53 (1.34)/*n* = 20	14.65 (1.93)/*n* = 21

**Table 4:** **Decline of the interaction effect gene by solar activity from the first trimester to birth on neuroticism (η^2^ are partial η^2^)**.

1. Trimester of gestation	*F*_(1, 245)_ = 4.14, *p* = 0.04; η^2^ = 0.017
2. Trimester of gestation	*F*_(1, 245)_ = 2.18, *p* = 0.14; η^2^ = 0.009
3. Trimester of gestation	*F*_(1, 245)_ = 2.83, *p* = 0.09; η^2^ = 0.011
Birth date	*F*_(1, 245)_ = 0.21, *p* = 0.65; η^2^ = 0.001

### Correlations between the personality dimension neuroticism and the volumes of hippocampus and amygdala

**Figure 5:**
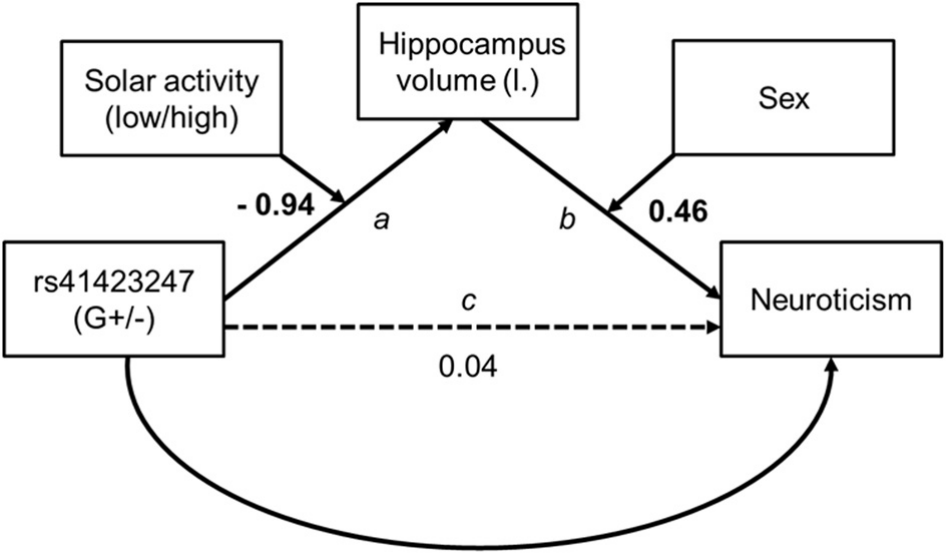
**Conditional model tested for indirect effects from genetics on personality**. Unstandardized path coefficients are shown. On path *a* and *b* the coefficients refer to the interaction term. Bold figures are significant (*p* < 0.01).

For the total sample no significant correlations could be observed between the volumes of the brain structures investigated and the personality dimension neuroticism. Given the influence of sex on both brain volumes and neuroticism, we decided to calculate the analysis of the neuroticism brain structure link additionally in both sex sub-samples. Interestingly, a robust inverse correlation between neuroticism and the left hippocampus volume could be observed in males (*r* = −0.35, *df* = 67, *p* = 0.003) but not in females (*r* = 0.13, *df* = 179, *p* = 0.11) after controlling for whole brain volume and age. Based on these findings it seemed worth to take a closer look at indirect effects by modeling previously reported effects in a global statistical model. Here, we aimed to find out if the hippocampus volume is a mediator between rs41423247 (G±) and neuroticism. Furthermore previous reported results in this result section suggested that solar activity moderated the effect between gene and hippocampus volume and sex the effect of hippocampus volume on neuroticism leading to a conditional effects model shown in Figure 5.

Model assumptions were met, indicated by significant moderation model summaries for path a [*F*_(3, 250)_ = 3.48, *p* < 0.05] and path b [*F*_(4, 249)_ = 3.74, *p* < 0.01]. Though there was no direct effect of rs41423247 (G±) on neuroticism all conditional indirect effects reached the level of significance and are shown in Table 5. In detail, the results revealed a “mediating” effect for male participants carrying the G− variant of rs41423247 and experiencing low solar activity during gestation through hippocampus volume on neuroticism (*B* = −0.14, *SE* = 0.08, 95% CI [−0.356, −0.028]) whereas for males with the G+ variant an indirect effect was observed for those participants with high solar activity [*B* = 0.18, *SE* = 0.10, 95% CI (0.034, 0.455)]. Conditional indirect effects for women were also statistically significant. But it has to be noted that confidence intervals for women were close to zero: Together with the absence of a correlation between hippocampus volume and neuroticism in women reported earlier, these results are a possible statistical artifact and therefore not interpreted. Furthermore, the model statistics backed up solar activity and sex as moderator as reported in the ANOVA models (see above). The interaction terms of solar activity by gene on hippocampus volume [*B* = −0.94, *SE* = 0.30, *p* = 0.002, 95% CI (−1.53, −0.35)] and hippocampus volume by sex on neuroticism [*B* = 2.49, *SE* = 0.71, *p* = 0.001, 95% CI (1.10, 3.88)] were statistically significant.

**Table 5:** **Conditional indirect effects of rs41423247 (G±) through left hippocampus volume on neuroticism**.

	**Low solar activity**				**High solar activity**			
	***B***	***SE***	***LLCI***	***ULCI***	***B***	***SE***	***LLCI***	***ULCI***
Female	0.05	0.03	0.002	0.145	−0.06	0.04	−0.185	−0.001
Male	−0.14	0.08	−0.356	−0.028	0.18	0.10	0.034	0.455

Coding gene variant: G− = 0, G+ = 1. Solar activity measured for the 1st trimester of gestation. Bootstrap samples = 1000. Level of confidence (bias corrected) = 95%.

As a last information in the result section the solar activity (and standard deviations) in the trimesters of pregnancy in the total sample and splitted up according to the allelic variants are presented in Table 6. Solar activity is presented in form of sunspot numbers.

**Table 6:** **Mean solar activity (and standard deviations) in the trimesters of pregnancy in the total sample and splitted up according to the allelic variants**.

	**Total sample**	**G+**	**G−**
1. Trimester	78.14 (56.09)	78.23 (55.51)	77.68 (59.72)
2. Trimester	77.40 (55.75)	77.12 (55.18)	78.89 (59.32)
3. Trimester	77.61 (57.25)	76.72 (56.70)	82.27 (60.52)

Solar activity is presented in form of sunspot numbers.

## Discussion

The present study investigated the influence of both molecular genetics and solar activity on the human brain structure. As hypothesized we could observe an interaction effect between rs41423247 and solar activity in the first trimester after conception: in detail, carriers of the G− variant showed the highest hippocampus volume in the left hemisphere in adulthood, but only when their mothers underwent pregnancy during a cycle of high solar activity. Interestingly, this effect was strongest for the first trimester. Afterwards, the effect declined towards birth. This is in line with observations by Dunkel Schetter and Glynn (2011) showing that environmental effects on neural development are particularly strong in this early phase of gestation. The left hippocampus represents the only brain structure under investigation, where this effect could be observed.

We are aware of the preliminary character of the present data (in need to be replicated by other work groups), but we are convinced of the validity of the results for several reasons. CC carriers of rs41423247 who gestated under the influence of high solar activity are not only associated with highest hippocampus volumes, but also with lowest neuroticism scores This fits nicely with the literature, as lower hippocampus volumes have been associated with depression (Sheline, 2011) and with high trait anxiety in personality research (Yamasue et al., 2008; DeYoung et al., 2010). In the introduction section we also mentioned the interesting findings by Knoops et al. (2010) linking higher evening cortisol/reduced suppression of cortisol in the dexamethasone test to lower hippocampus volumes. These studies are of interest, because we aimed to bridge the link from molecular genetics (with a focus on a gene coding for the glucocorticoid receptor) via brain structure on personality.

In the context of the personality-brain-structure-link, we observed a robust negative correlation between neuroticism and the left hippocampus volume explaining about 10% of the shared variance. But: This effect could only be detected in our male participants. In general, sex always needs to be considered as an important nuisance variable when dealing with structural data of the human brain (Hu et al., 2011). Fitting to the present data, we were able to demonstrate in previous work, that fractional anisotropy values of white matter tracts can be linked to the male but not female anxious personality (Montag et al., 2012). This study is mentioned here (although white matter has been investigated), because the temporal lobe was the focus of this earlier study. Among others white matter tracts linking the hippocampus and the cingulum were investigated here. A meta-analysis by Wager et al. (2003) revealed that the male compared to female brain is more lateralized with respect to emotionality, which also could explain our findings. In general, this warrants more attention in future research, because other work groups found hippocampus associations with anxiety in both males and females (Yamasue et al., 2008; DeYoung et al., 2010). A new review as well as own data by our group on anxiety-related personality traits and the structure of the human brain outlines the heterogeneity of the studies in the field in detail (Liu et al., 2013; Montag et al., 2013b). In sum, the here mentioned approaches to explain the present data have to be viewed cautiously.

In a further step, the significant results were integrated in a path model showing an indirect effect from gene through brain structure on self-reported behavior for men. The indirect effect could also explain the reported interaction effect of gene × solar activity on neuroticism, which was not included in the tested model. The model results imply that the solar activity is necessary to affect rs41423247's indirect impact on neuroticism through hippocampus volume. Furthermore, the model explained about 5% of variance in neuroticism. This is in line with our previous findings of explained variance in neuroticism by left hippocampus volume.

How can the present findings be explained from a molecular perspective? The investigated SNP on the gene coding for the glucocorticoid receptor has been demonstrated to be an important modulator of the HPA-axis in the context of negative emotionality (Wüst et al., 2004). Therefore, it is of course a very likely target for research in the psychological/psychiatric neurosciences. Given the literature on the NR3C1 gene and depression as well as on the HPA-axis, it is not surprising that this SNP can be linked to the hippocampus structure and the personality dimension neuroticism. What is surprising though, is that no main effect could be observed, but only an interaction with high solar activity in the first trimester. Although highly speculative, carriers of the CC variant could experience an inhibition of the developing HPA axis because of the high solar activity in the first trimester of their life after conception. This special constellation between genetic make-up and solar activity might reflect an early resilience factor for developing psychopathological disorders in adulthood in form of neural growth in the hippocampus (at least in the left hemisphere). Given the lack of knowledge in the field of solar activity and gene expression we would like to refrain from further speculations at this point.

The present study aims to introduce solar activity together with molecular genetic variables as potential modulators of brain structure and personality to neuroscientificly oriented psychological and psychiatric research. This is in tradition with studies as by Caspi et al. (2003) investigating gene by environment interaction effects on psychological phenotypes. As replication studies are of particular interest in the field of molecular genetics and brain imaging—especially when dealing with such new ideas as solar activity—we would like to encourage researchers to include this easily calculated measure of solar activity in their future research. Moreover, future research endeavors might also investigate the here reported effects on a more “direct” molecular genetic level including epigenetics.

There are several limitations to the present study: First of all, recent results by Velders et al. (2012) yielded evidence that in the unborn child, rs41423247 interacts with maternal psychological health on emotional behavior and cortisol reactivity of the then born child at the age of three. At first sight this strongly supports our present findings. Interestingly, Velders et al. (2012) reported in contrast to the findings by Van Rossum et al. (2006) and our findings that the C allele in combination with maternal psychological symptoms predicts emotional problems of the children at age of three. Given that these authors also investigated an interaction effect between rs41423247 and environmental influences on psychological health of the unborn child in later life (here age three), this contrary finding is especially noteworthy. Although these findings seem inconsistent at first sight, we'd like to refer to the idea that solar activity might represent a total different environmental “stressor/influence” compared to maternal psychological problems and could therefore interact in other ways with rs41423247 leading to other psychological outcomes. A second limitation represents missing information in the present study: Unfortunately, we did not ask our participants if they were preterm or late-term born. This could affect our calculated date of conception. Given our large sample under investigation, however, chances are that such bias is negligible because it should average out across the whole sample. Moreover, when doing the analysis with another time window (using quartiles instead of trimester) the observed results for the first quartile are similar. Although counting sunspot numbers is an easy way to assess solar activity it is not the only way. Future studies addressing underlying mechanisms of solar activity should also address different kinds of indicators for solar activity or weak magnetic field changes on earth such as the Kp Index (Rangarajan and Barreto, 1999). Furthermore, our conditional effects model represents one approach to analyze the data. Different statistical approaches might yield slightly different results, here. Two more limitations need to be mentioned. First: As our main results focus on interactions effects, the cell sizes of our study design are in parts very small (*n* = 20 and *n* = 21). Therefore, even larger samples sizes are needed in the future to overcome this issue. Second: A last important issue to be mentioned concerns the solar activity itself: As mentioned in the introduction Davis and Lowell (2004) suggested that changes in UVR during the solar cycle might increase polygenic mutations in humans potentially leading to a higher occurrence of major mental illnesses. But UVR cannot penetrate into skin deeper than 1 mm (Bruls et al., 1984; cited after Diffey, 1991). Therefore, a direct influence of solar activity on the unborn child is very unlikely. Nevertheless UVR could initiate physiological and behavioral adaptions in the mother during gestation, therefore describing an indirect mechanism. Moreover, the reader should note that UVR intensity is dependent on a lot of factors, e.g., clouds, angle of the sun and obstructions such as buildings or clothes (Diffey, 1991). As a consequence, weak magnetic field changes initiated by the solar activity could be more interesting to explain the underlying mechanism behind the here reported results.

In sum, the investigation of genes together with solar activity might represent a noteworthy research avenue in the future for the investigation of psychological and psychiatric research questions.

### Conflict of interest statement

The authors declare that the research was conducted in the absence of any commercial or financial relationships that could be construed as a potential conflict of interest.

## Footnotes

^1^Also referred to as Wolf- or Zurich-number: *R* = *k*(10 × *g* + *s*); *k* = observation factor, *g* = sunspot groups >10, s = individual sunspots visible on the solar disk.

^2^Given the small literature with respect to solar activity on gene expression, we refrain from a directed hypothesis here.

^3^Solar Influences Data Analysis Center (SIDC): http://sidc.oma.be/

^4^*Mj* = (*a*1*j* + *a*4*jW*) (*b*1*j* + *b*4*jV*). *W* = moderator path a. *V* = moderator path *b*. *a*4 = *XW*. *b*4 = *MV*.
